# Impact of Patient-Specific Hip Joint Geometry on the Morphology of Acetabular Fractures

**DOI:** 10.3390/jcm13237332

**Published:** 2024-12-02

**Authors:** Amadeo Touet, Yannick Schmiedt, Jessica Köller, Christian Prangenberg, Davide Cucchi, Kristian Welle, Christoph Endler, Sebastian Scheidt

**Affiliations:** 1 Clinic for Orthopedics and Trauma Surgery, University Hospital of Bonn, 53127 Bonn, Germany; 2 Clinic for Diagnostic and Interventional Radiology, University Hospital of Bonn, 53127 Bonn, Germany

**Keywords:** acetabulum, fracture, femur head, proximal femur, biomechanics, trauma mechanisms, computed tomography

## Abstract

**Background**: Acetabular fractures continue to pose a major challenge in clinical practice, not least because of the growing geriatric population. While the influence of the force vectors on fracture formation is well established, the impact of anatomical factors on fracture morphology remains poorly understood. The aim of this study was to investigate patient-specific hip joint geometry, identify structural risk factors and correlate these with the resulting fracture patterns. **Methods**: This retrospective cohort analysis included 226 patients (Mdn age = 58 yrs.) with acetabular fracture categorized by Judet/Letournel and the AO/OTA classification. Computed tomography (CT) datasets of the injured and contralateral sides were analyzed using multiplanar reconstruction. Parameters included modified center-edge (CE) angle (Wiberg), rotation angles (Ullmann and Anda), acetabular sector angle (Anda), true caput-collum-diaphyseal (CCD) angle, femoral head diameter and volume, as well as femoral neck length, circumference, and diameter. In addition, intrarater reliability within a subcohort was assessed for the metric measurements and inter-rater analysis for the classification of the entire sample. **Results**: The primary analysis showed direct effects of femoral head diameter, femoral neck length and femoral head size on the fracture type according to AO/OTA (type A/B/C), whereby this effect was particularly seen between type A and type C fractures (*p* = 0.001). Ordinal regression identified femoral head diameter as the only significant predictor (*p* = 0.02), with a 25% increased likelihood of complex fractures per unit of change. Low-energy trauma doubled the risk of severe fractures. Specific findings include a higher acetabular anteversion in anterior column fractures. Age correlated positively with the cause of injury and fracture type. The inter-rater reliability for fracture classification was excellent, as was the intrarater reliability of the measurements. **Conclusions**: This study suggests that anatomical factors, particularly proximal femoral geometry, have an impact on acetabular fracture morphology—in addition to factors such as trauma type and patient demographics.

## 1. Introduction

Fractures of the acetabulum pose a significant challenge in clinical practice, characterized by a diverse patient population with varying mechanisms of injury [1,2,3]. These fractures can result from both high-energy trauma, like motor vehicle accidents or falls from great heights, as well as low-energy trauma, such as a simple standing fall. Understanding the biomechanical and structural parameters that influence fracture formation is critical to optimize treatment algorithms and patient outcomes. Basically, those fractures occur when external forces drive the femoral head into the acetabulum. In this context, factors such as trauma kinematics, bone quality and the position of the femur during the impact play a decisive role in the formation of acetabular fractures [4]. Dakin et al. have shown a clear correlation between acetabular fractures and trauma mechanisms and emphasized the importance of looking at force vectors in fracture analysis [5]. Considering the four types of force transmission described by Letournel et al.—via the greater trochanter, via the knee in 90° knee flexion, via the foot in knee extension and via the dorsal pelvis—typical fracture patterns could be determined depending on the position of the femur during the impact [6].

The assessment of acetabular fractures still relies on the column principle described by Rouvière and the subsequent established classification by Judet and Letournel in the early 1960s [7,8]. According to Judet/Letournel, fractures are categorized into five elementary and five associated fracture types, providing valuable information for further therapy and particularly the appropriate surgical approach. This anatomically based classification is still widely used and relevant in today’s clinical practice [9]. Following the principles of Judet/Letournel, the alphanumeric (AO/OTA) Arbeitsgemeinschaft für Osteosynthesefragen/Orthopedic Trauma Association consensus classification was developed, which includes prognostically relevant information [10]. According to AO/OTA classification, fractures are categorized into three fracture types: Type A—one column involved, partial articular fracture; Type B—both columns involved, partial articular fractures; and Type C—both columns involved, complete articular fractures. Moreover, there is a subdivision into further fracture groups (1–3), subgroups (1–3) and additional qualifiers [4].

While the influence of anatomical factors on proximal femur fractures has been well studied, the effects on acetabular fractures are still poorly understood [11,12]. Therefore, the aim of this study is to investigate the patient-specific hip joint and acetabulum geometry, detect structural risk factors, and correlate them with the resulting acetabular fracture patterns. To our knowledge, this study is the first to examine this direct relationship in a cohort of patients with high- and low-energy trauma events in direct correlation with fracture classification.

## 2. Methods

### 2.1. Patient Selection Process

**Figure 1:**
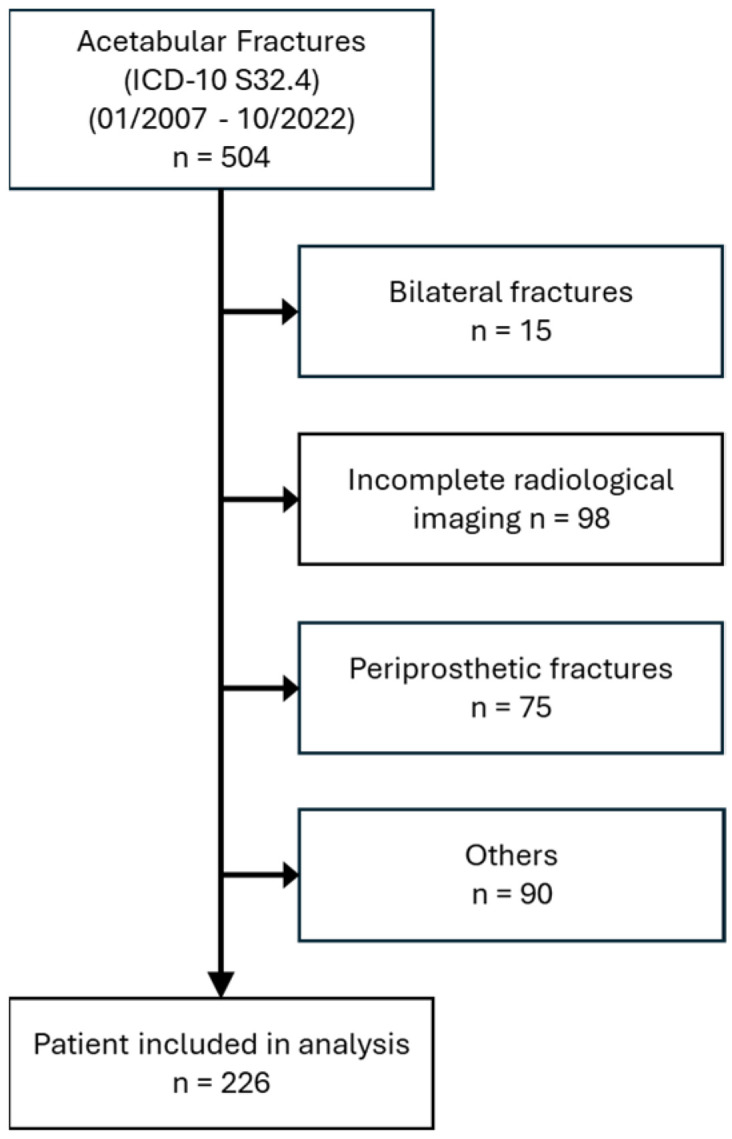
Patient selection process.

This retrospective study included a total of 226 patients with traumatic acetabular fractures who were treated in our university level 1 trauma center between 01/2007 and 10/2022. The cohort consisted of 74 female (32.7%) and 152 male (67.3%) patients with a median age of 58 years (42/74). Considering the primary endpoint of this study, we included patients over 18 years with acetabular fractures resulting from either low- or high-energy trauma. Exclusion criteria were concomitant femoral fractures, hip arthroplasty on both the ipsilateral and contralateral side and other foreign material, additional hip and pelvic pathologies (e.g., grade IV° osteoarthritis, tumors and pre-existing fractures) and incomplete radiological imaging. The patient selection process is illustrated in Figure 1. This study was approved by the local Ethics Committee (RN 004/23) and informed consent was obtained from patients for the anonymous use of their clinical data for retrospective analysis.

### 2.2. Fracture Classification

The fractures were classified according to Judet/Letournel, based on their elementary (posterior wall, posterior column, anterior wall, anterior column and transverse) and associated (T-shaped, posterior wall with posterior column, posterior wall with transverse, anterior column or wall with posterior hemitransverse and both columns) fracture types. A sample-specific distribution is shown in Table 1. However, some subgroups were relatively small, so an alternative classification had to be chosen for a more robust statistical analysis. Therefore, an allocation was made according to the AO/OTA Fracture and Dislocation Classification. With comparable problems, the final fracture classification focused on the universal major AO/OTA fracture types. The distribution yielded AO/OTA type A = 127, AO type B = 21, and AO type C = 78.

### 2.3. Measurement of Proximal Femur Geometry and Acetabular Geometry

The graphical evaluation and measurement were made with “Deep Unity Diagnost” software (DeepUnity Diagnost 1.1.0.0 ©, Dedalus HealthCare, Bonn, Germany, 2021) using thin-slice computed tomography (CT) images (slice thickness < 1.5 mm). Utilizing multiplanar reconstruction, we achieved a comprehensive three-dimensional visualization that aligned the pelvis in space with reference to key landmarks such as the pubic symphysis, the anterior superior iliac spine and the sacroiliac joints. This approach enabled accurate measurements of the CT data sets.

The acetabular geometry measurements were performed on the healthy contralateral side. In the coronal plane, the modified center-edge (CE) angle according to Wiberg (°), which describes the position of the femoral head in relation to the acetabulum, and the modified rotational angle according to Ullmann (°), assessing acetabular rotation, were measured. In the transversal plane, the acetabular anteversion (°) was measured according to the technique of Anda et al. [13]. To quantify the femoral head coverage, the anterior acetabular sector angle (AASA; °) and the posterior acetabular sector angle (PASA; °) as well as the sum of these two values (HASA; horizontal acetabular sector angle; °) were assessed [14].

**Figure 2:**
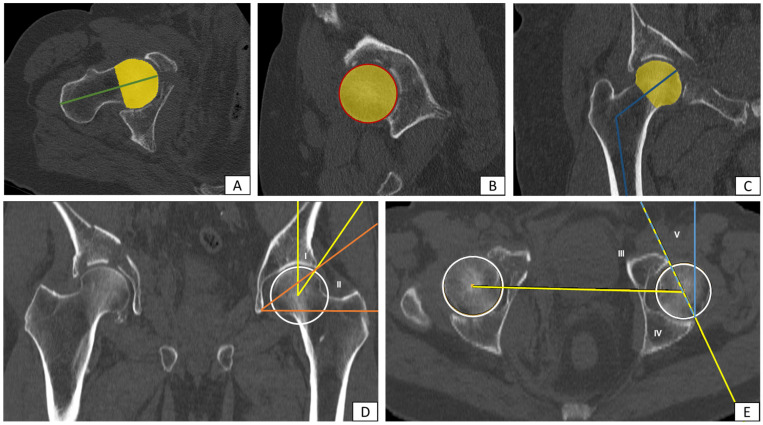
Example of the measurement of proximal femur and acetabular geometry using CT data with multiplanar reconstruction: (**A**) femoral neck length (green), (**B**) femoral head diameter (red), (**C**) real CCD angle (blue), (**A**–**C**) femoral head volume (yellow), (**D**) (I) CE angle mod. acc. to Wiberg (yellow), (**D**) (II) rotational angle mod. acc. to Ullmann (orange), (**E**) (III) anterior acetabular sector angle (yellow/AASA), (**E**) (IV) posterior acetabular sector angle (yellow/PASA), (**E**) (V) acetabular anteversion acc. to Anda (blue).

In contrast, the examination of the femoral geometry was carried out on the fractured side. The multiplanar reconstruction allowed precise alignment of the femur, facilitating accurate measurements. In the style of native radiological measurement, the adapted real caput-collum-diaphyseal (CCD) angle (°) was determined. Several other parameters such as femoral head diameter (mm), femoral neck length (mm), femoral neck circumference (mm) and femoral neck diameter (mm), both longitudinal and transverse oval, were also assessed. The femoral head volume (mm^3^) was obtained by manually labelling the structures as “region of interest” (ROI) in each CT slice and automated volume output. An example of the CT-based measurements is shown in Figure 2.

Alongside the primary research question, a more detailed analysis of trauma kinematics (low- or high-energy trauma) was performed.

### 2.4. Reliability

To assess the measurement reliability, intrarater reliability was tested on a subgroup (*n* = 30), selected by a simple random sample using a number generator in SPSS (IBM SPSS Statistics for Windows, Version 27.0. Armonk, NY: IBM Corp, Armonk, NY, USA, 2020). The measurements were then repeated by the same rater after 14–21 days. For the Judet/Letournel and AO/OTA classifications, inter-rater reliability testing was performed for the entire sample, with a second independent rater classifying the sample to assess consistency.

### 2.5. Statistics

The collected data were entered into Microsoft Excel (Microsoft Corporation, Redmond, DC, USA, 2018) and transferred to SPSS for further analysis. Given a nonparametric distribution of the data, a one-way ANOVA on ranks was performed to directly compare the fracture types, followed by a post hoc test using the Dunn–Bonferroni method. Ordinal regression was used to model the dependence of the identified parameters in relation to fracture types. Furthermore, the Mann–Whitney U test (MWU) and subsequent logistic regression model were calculated for the analysis between low- and high-energy trauma. The exact Fisher’s test (FET) was utilized for gender-specific analysis. A two-way mixed intraclass correlation coefficient (ICC) was used to assess intrarater reliability, while Kendall’s tau was used to test inter-rater reliability. In view of the distribution, the median (Mdn) is reported with the interquartile range (IQR). Overall, a significance level of 0.05 was applied.

## 3. Results

**Figure 3:**
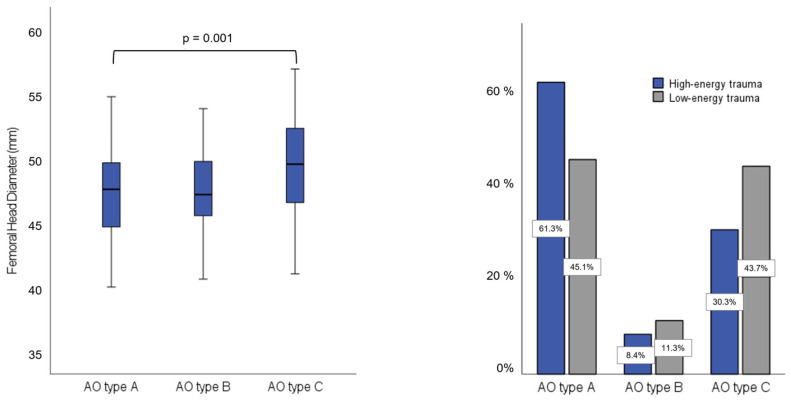
(**left**) Femoral head diameter for the three main fracture types (A/B/C) according to the AO/OTA classification. The Dunn–Bonferroni post hoc test shows a significant difference between type A (Mdn. 47.6 mm (44.6/49.6)) and type C (Mdn. 49.5 mm (46.6/52.2) (*p* = 0.001)). (**right**) Distribution of the trauma-energy mechanism (low-energy: grey, high-energy: blue) for the three main fracture types (A/B/C) according to AO/OTA classification, with percentages shown for each group.

Using the one-way ANOVA on ranks, the parameters femoral head diameter, femoral head size and femoral neck length showed a significant effect on the fracture type according to AO (AO type A/B/C) (Table 2). For all mentioned parameters, however, this effect could only be shown in the post hoc test between AO Type A and Type C (e.g., femoral head diameter; z = −3.57; *p* = 0.001; Figure 3). Especially when considering the cause of injury separately (high-energy trauma *n* = 155 and low-energy trauma *n* = 71), the correlation outlined above is particularly evident for patients after high-energy trauma. Using the regressions model and considering the covariates, only the femoral head diameter was a positive and significant predictor (*p* = 0.02, Exp (B) = 1.25) (Table 3). If the femoral head diameter increases by one unit, the relative likelihood of sustaining a more severe fracture type according to AO/OTA classification increases by 25%.

Acetabular parameters were found to have no significant effect on the fracture morphology according to the AO/OTA fracture types in this cohort. However, there was a notable difference in acetabular anteversion between fractures involving the anterior and posterior column. Specifically, fractures involving the anterior column had a higher acetabular anteversion angle than fractures involving the posterior column (20.25° (15.5/24.65) vs. 16.6° (14.5/21.0); *p* = 0.028)). Additionally, we observed a gender-specific difference in the anteversion of the acetabulum, with men showing a significantly lower anteversion angle compared to women (MWU; U = 3780.0; *p* < 0.001; r. acc. Cohen = 0.3). Integrating these findings, we analyzed that men are more likely to sustain fractures with isolated posterior involvement (post wall, post column, and post wall and column), whereas women are more likely to experience anterior fractures of the anterior wall and column (FET; *p* = 0.019).

From the regression analysis, the trauma energy mechanism also emerges as a relevant parameter (*p* = 0.017)—patients with a low-energy trauma have twice the risk of suffering a more severe AO fracture type (Table 2; *p* = 0.017, Exp (B) = 0.503). A comparable result was demonstrated with regard to the mechanism of trauma energy between elementary and combined fracture types (Judet/Letournel) using the Mann–Whitney U test (*p* = 0.008; Z = −2.66). Patients after high-energy trauma tend to exhibit elementary fracture types, while low-energy trauma is more likely associated with combined fracture types. In this context, it is important to emphasize the positive correlation between the parameter age and the cause of injury (*p* < 0.001; Z = −7.54) as well as with the AO fracture types (*p* = 0.014). Older patients were more likely to experience a low-energy trauma and were more likely to have severe AO fracture types in terms of fracture morphology.

Using ROC analysis and the Youden index, an age of 53.5 years was determined as the cut-off value in relation to the trauma-energy mechanism (high- vs. low-energy trauma; AUC 0.813; *p* < 0.001). Thus, 91.5% (Sens.) of patients after high-energy trauma were under 53.5 years old. In terms of patient demographics, a positive effect was found between both weight and BMI and the resulting fracture pattern. Higher weight tends to be associated with more complex/associated fracture types. Excellent inter-rater reliability was demonstrated for the allocation to the respective fracture types (*n* = 226; *p* < 0.001; Kendall Tau-b = 0.99). The subcohort analysis (*n* = 30) also showed a very good intrarater reliability with an ICC range from 0.893 to 0.998 (Supplementary Materials).

## 4. Discussion

Understanding the morphology of acetabular fractures and their underlying influencing factors is crucial for improving treatment algorithms and optimizing patient outcome. However, there have been very few studies looking at the anatomical factors that may influence acetabular fractures [15,16,17]. Therefore, this study primarily examined the impact of proximal femur and acetabulum geometry on fracture morphology, categorized according to the major AO/OTA classification fracture types. Overall, we observed that, in the present patient cohort, only proximal femoral geometry, particularly femoral head diameter, had a relevant influence on the resulting fracture type according to AO/OTA. Furthermore, we observed a positive correlation between the acetabular anteversion and anterior column injuries, but we could not determine any statistical influence of the acetabular geometry on the major fracture types (A/B/C) according to the AO/OTA classification.

Our findings suggest that, from an anatomical perspective, the size of the femoral head plays a decisive role in fracture formation. Larger femoral heads may lead to greater biomechanical stress and force transmission to the acetabulum during trauma, resulting in more complex fracture patterns. Even though the regression analysis only showed an effect for the femoral head diameter parameter, a similar trend was observed for the femoral head volume using one-way ANOVA on ranks. Possible methodological causes are discussed in detail later on. The force applied to the hip joint, with stress defined as a vector quantity per unit area, is primarily influenced by the functional surface as the contact between the femoral head and acetabulum. The concept of “physiological incongruity” refers to the mismatch between the size of the femoral head and the acetabulum, impacting load distribution within the joint. This incongruity is particularly notable in younger individuals and tends to reduce with age and the associated degeneration of joint structures [18,19,20,21]. When examining the load-bearing zones of the hip and considering subchondral mineralization, Müller-Gerbl et al. demonstrated that younger individuals typically exhibit higher density values in the dorsoventral regions of the acetabulum. In contrast, older patients show increased density primarily in the central region [19]. These differences in mineralization patterns and subsequently load-bearing zones could lead to a more centralized force induction during trauma in older patients, which, from an anatomical point of view, could explain the more complex fracture types involving both acetabular columns in this patient group. This hypothesis is supported by the fact that the subchondral bone becomes denser with increasing age and loses its inherent elasticity [19,20].

None of the other parameters of proximal femur geometry showed any significant effects on fracture morphology in the analysis performed. Some adjustments were made to the measurement methodology. According to Wiberg, the CE angle is a parameter measured in native X-ray images [22]. However, in accordance with comparable studies, it was adapted for CT imaging to enable assessment in the frontal plane [23]. Gebre et al. observed a lower CCD angle (neck shaft angle/NSA) of 122.1° in patients with acetabular fractures after low-energy trauma compared to 124.6° in the control group [15]. In our study, the CCD angle, which was originally also described for native radiographs, was measured using CT imaging to avoid potential rotation measurement errors in anteroposterior X-ray images. This approach enables more accurate measurements through multiplanar reconstruction. The median real CCD angle in our study was 118° (114°/122°), which is lower than reported in other studies, whereat the different measurement methods must be considered. Nevertheless, we did not confirm the effect described by Gebre et al. in our study. Instead, we found a significant negative correlation between age and the CCD angle. Older patients tend to have a smaller CCD angle, which Boymans et al. describe as a shift towards a relatively varus position [24]. This raises the question of whether the correlation described by Gebre et al. is caused by the CCD angle or whether the independent variable “age” significantly influences the result. Moreover, our study showed that the variable “age” has a strong influence on both the type of trauma and the resulting fracture morphology. Older patients showed more severe fracture types according to AO/OTA (*p* = 0.014) and, of course, there was a clear correlation between age and the trauma energy mechanism (low- and high-energy).

In the assessment of acetabular fractures, distinguishing between low-energy and high-energy trauma is essential. These varying injury mechanisms are associated with altered biomechanics and often result in different fracture patterns. Depending on the impact, fractures of the posterior acetabular wall are frequently observed in high-energy trauma [16,25,26]. Gänsslen et al. reported that over 90% of these fractures occur in the context of high-energy trauma, with dashboard injuries being particularly prominent [26]. Similarly, Kim et al. identified motor vehicle accidents as a significant predictor of posterior wall fractures [16]. In contrast, older patients who experience low-energy trauma tend to have injuries involving the anterior acetabular column [25]. Primarily, this is related to the trauma mechanism of a fall onto the side and the subsequent force transmission via the greater trochanter [2]. Previous studies primarily focused on low-impact fractures in the geriatric setting [15]. However, the incidence of geriatric high-energy trauma is increasing significantly due to demographic change and longer participation in everyday life [2,3]. Our study also showed that several geriatric patients were exposed to high-energy trauma, with 17.4% of patients aged 70 or older falling into this category. This patient group is associated with a higher overall complication rate and 30-day mortality [27]. Overall high-energy trauma predominated in our patient cohort, accounting for almost two thirds of cases. Most of these accidents were traffic accidents involving cars or trucks (33%), motorcycles (14%), and falls from heights greater than three meters (30%). Due to the retrospective nature of data collection and extraction from emergency medical protocols and records, a clear differentiation between the trauma energy mechanism was not always possible. Furthermore, there is no clear definition in the literature for categorizing these types. Ultimately, our assignment relied on a combination of available literature and clinical judgement [3,25,27,28]. Notwithstanding this, we were able to determine an age cut-off of 53.5 years for the two trauma mechanisms using the ROC analysis. This goes in line with comparable studies such as that by Goyal et al., which set a cut-off at 55 years [29]. In the context of age, it is important to consider bone quality and osteoporosis when examining anatomical factors and fracture formation, especially given the increasing proportion of geriatric patients in the clinical setting [25]. The impact of bone quality on proximal femur fractures is well known [30]. Likewise, studies have found a correlation in acetabular fractures. Gebre et al. found that lower “bone mineral density” in the acetabulum and femoral head, together with altered trabecular structure, are significant risk factors for low-energy fractures [31]. In the present study, no additional bone density measurements could be performed due to the retrospective design. Furthermore, alternative methods that quantify Hounsfield units in CT imaging and correlate with bone density could not be used due to the lack of standardized CT imaging protocols [32,33].

Regarding the primary endpoint of this study, we assessed the acetabular geometry on the contralateral side, assuming a strong correlation and reproducibility [15]. The previously mentioned correlation between acetabular version in the axial plane and anterior column injuries has also been demonstrated by Werner et al. [17]. Notably, the anterior wall is more rigid than the posterior wall [34]. Furthermore, we found no correlation between acetabular geometry and the three major AO/OTA fracture types (A/B/C). In a retrospective analysis of 107 patients, Kim et al. identified the acetabular index (AI) as an index of acetabular coverage and the acetabular depth-to-width ratio (AD/WR) as significant risk factors for posterior wall fractures (PW) [16].

We have found that patients with higher weight/BMI tend to have more severe fractures, especially those involving both columns, a cohort which is already characterized by an increased rate of perioperative complications [35].

The results of this study must be discussed in the context of the chosen methodology. For the radiological evaluation, CT imaging was used, as it is superior for the classification and assessment of acetabular fractures [36,37]. Multiplanar CT reconstruction provides higher diagnostic accuracy compared to radiographs or traditional 2D-CT imaging [38]. In addition, this technique allows individualized alignment, overcoming varying positioning of patients, as it is common in the polytrauma-CT setting. This aligns with the 3D-visualization approach, a concept that has become increasingly important in recent years. It does justice to the complex configuration of the pelvis and acetabulum, supports the interpretation and understanding of the fracture and, thus, helps in the decision-making process [39,40,41].

The primary classification of fractures continues to follow the established anatomical Judet/Letournel classification. The most common fracture type observed in our study was the two-column fracture, which is consistent with the results of various systematic reviews [42,43]. However, our study showed a shift in the distribution of fracture types compared to other established studies [1,7,36,44]. In Letournel’s 1964 study, posterior wall fractures were described as the most common fracture type, whereas our results show a higher incidence of fractures of the ventral acetabulum [7]. The reasons for this discrepancy are unclear, but three important aspects need to be discussed. First, our study examined a heterogeneous group of patients with a higher average age in comparison to other studies. For instance, Porter et al., whose study had a similar fracture distribution to the Juet/Letournel cohort, reported an average age of 36 years for patients who primarily experienced high-energy trauma [44]. This contrasts with the significantly higher age in our study. Considering the previously discussed aspects of trauma etiology and age-dependent injury patterns, this may explain the difference. This is supported by Albrektsson et al., who noted a relevant shift in fracture dislocation towards more anterior wall injuries in older patients experiencing low-energy trauma [1]. Second, there is a potential selection bias in our patient cohort, explained by the large trauma center with a wide catchment area. As a result, there is a high proportion of secondary transfers from other hospitals, which generally involves patients requiring surgical interventions. Third, discrepancies in the applicability of the classifications could also contribute. Hutt et al. [36], in their analysis of 100 acetabular fractures, found 35% of cases to be “unclassified”, whereas our study applied a “best match” classification approach. Given a robust statistical analysis, the AO/OTA fracture types were used as final classification. However, it is important to note that those major fracture types only partially reflect the severity of the fractures. For instance, T-fractures categorized as type B according to AO/OTA may be more complex in terms of severity and prognosis than some two-column fractures (type C). Moreover, the modifiers of the AO/OTA classification, which provide prognostic information, could not be considered due to subgroup issues. Despite this, the small sample size of type B fractures must be evaluated critically for statistical purposes.

Besides the previously reported limitations of the study, the retrospective design must be emphasized. The inclusion of both low- and high-energy trauma results in a heterogeneous patient cohort, which nonetheless reflects everyday clinical practice in a level I trauma center. In terms of anatomical parameters, measuring the femoral torsion would have been interesting. However, this was not possible in view of the used imaging modality, as CT scans usually do not include the distal femur. Additionally, considering the pelvic tilt would have been beneficial for a more comprehensive analysis of anatomical parameters. As described above, the role of the femoral position at the time of impact plays a fundamental role in fracture formation. Given the retrospective design, it is difficult to provide these data. Regarding the primary endpoint of this study, it must be noted that the articulation between the femoral head and acetabulum involves a complex interaction of forces and plastic deformations of the anatomical structures. This complicates modelling efforts, as it is not simply a static equilibrium, but a dynamic process influenced by various factors. It is important to acknowledge that assessing anatomical factors alone is insufficient for understanding fracture formation. It is far more important to evaluate these parameters within the context of dynamic formation process and patient-specific factors. Further research should focus on biomechanical analysis to refine clinical decision-making in acetabular fracture management.

## 5. Conclusions

In summary, this study highlights the importance and influence of patient-specific anatomical factors on the morphology of acetabular fractures. Key findings include that a larger femoral head diameter correlates with more complex acetabular fracture patterns, while increased acetabular anteversion is associated with fractures involving the anterior column. Additionally, a correlation between the severity of the acetabular fracture and the trauma mechanism could be established. Understanding these factors is essential for personalized treatment planning and improving patient outcomes.

## Figures and Tables

**Table 1:** Distribution of fracture types according to Judet/Letournel.

Fracture Types		*n*	%
Elementary fractures	Posterior wall	12	5.3
	Posterior column	5	2.2
	Anterior wall	34	15.0
	Anterior column	70	31.0
	Transverse	7	3.1
Associated fractures	T-shaped	9	4.0
	Posterior wall with posterior column	6	2.7
	Posterior wall with transverse	3	1.3
	Anterior column/wall with posterior hemitransverse	2	0.9
	Both columns	78	34.5
		**226**	**100.0**

**Table 2:** Analysis of femoral and acetabular parameters using one-way ANOVA on ranks, presented as median (Mdn.) and interquartile range (IQR). The statistically significant values are marked with an *.

Parameter	Mdn.	IQR	*p*-Value
Femoral head diameter (mm)	48.2	45.3/50.7	0.001 *
Femoral neck length (mm)	101.0	95.2/106	0.017 *
Femoral head volume (mm^3^)	49.7	39.5/56.9	0.018 *
Femoral neck diameter (mm)	34.0	31.7/36.3	0.083
Femoral neck circumference (mm)	112.0	103.9/119	0.057
Real caput-collum-diaphyseal (CCD) angle (°)	117.9	114.3/122	0.113
Mod. center-edge (CE) angle (Wiberg; °)	37.7	32.8/42.7	0.980
Rotational angle mod. (Ullmann; °)	38.0	34.9/40.5	0.476
Acetabular anteversion (Anda; °)	19.4	15.4/23.7	0.341
Femoral head coverage (Anda; °)			
Anterior acetabular sector angle (AASA)	64.1	58.3/70	0.178
Posterior acetabular sector angle (PASA)	104.9	96.6/112.2	0.570
Horizontal acetabular sector angle (HASA)	168.4	157.8/180	0.245

**Table 3:** Ordinal regression analysis modeling the dependence of identified parameters on fracture types, with (B) indicating the predictor’s coefficient and Exp (B) representing the odds ratio for a one-unit increase in the predictor. The statistically significant values are marked with an *.

Parameter	(B)	*p*-Value	Exp (B)
Femoral head diameter (mm)	0.223	0.020 *	1.250
Femoral neck length (mm)	−0.004	0.909	0.996
Femoral head volume (mm^3^)	−0.034	0.261	0.966
Trauma energy mechanism (high vs. low)	−0.688	0.017 *	0.503

## Data Availability

The data presented in this study are available on request from the corresponding author due to privacy.

## Supplementary Materials

The following supporting information can be downloaded at: https://www.mdpi.com/article/10.3390/jcm13237332/s1, Table S1: Intrarater reliability analysis of the subgroup (*n* = 30), selected through simple random sampling using SPSS (IBM SPSS Statistics for Windows, Version 27.0, Armonk, NY: IBM Corp., Armonk, NY, USA, 2020). The table presents the intraclass correlation coefficients (ICC), 95% confidence intervals (CI) and the standard error of measurement (SEM) for the respective parameters, comparing initial measurements with those repeated after 14–21 days.
