# Supplementary material for: Impact of Patient-Specific Hip Joint Geometry on the Morphology of Acetabular Fractures
Source: J Clin Med. 2024 Dec 2;13(23):7332. doi: 10.3390/jcm13237332 (PMC11642643; doi:10.3390/jcm13237332)
Supplement: Supplementary file 1 [file jcm-13-07332-s001.zip › jcm-3133021-supplementary.pdf]

Supplementary File

Table S1. Intrarater reliability analysis of the subgroup (*n* = 30), selected through simple random sampling using SPSS (IBM SPSS Statistics for Windows, Version 27.0, Armonk, NY: IBM Corp, Armonk, NY, USA, 2020). The table presents the intraclass correlation coefficients (ICC), 95% confidence intervals (CI) and the standard error of measurement (SEM) for the respective parameters, comparing initial measurements with those repeated after 14-21 days.

Parameter	ICC	CI	SEM
Femoral head diameter (mm)	0.990	0.978/0.995	0.732
Femoral neck length (mm)	0.998	0.996/0.999	0.672
Femoral head volume (mm^3)	0.998	0.996/0.999	0.969
Femoral neck diameter (mm)	0.984	0.965/0.992	0.965
Femoral neck circumference (mm)	0.993	0.986/0.997	2.008
Real caput-collum-diaphyseal (CCD) angle (°)	0.893	0.770/0.951	3.179
Mod. center-edge (CE) angle (Wiberg; °)	0.952	0.897/0.978	2.342
Rotational angle mod. (Ullmann; °)	0.955	0.903/0.979	1.659
Acetabular anteversion (Anda; °)	0.956	0.905/0.980	3.020
Femoral head coverage (Anda; °)			
Anterior acetabular sector angle (AASA)	0.995	0.990/0.998	2.152
Posterior acetabular sector angle (PASA)	0.993	0.985/0.997	2.422
